# Time-Course Transcriptomic Profiling of Floral Induction in Cultivated Strawberry

**DOI:** 10.3390/ijms23116126

**Published:** 2022-05-30

**Authors:** Jiahui Liang, Jing Zheng, Ze Wu, Hongqing Wang

**Affiliations:** 1 Department of Fruit Science, College of Horticulture, China Agricultural University, Beijing 100193, China; jiahuiliang1230@163.com (J.L.); zj49911228@126.com (J.Z.); 2 Key Laboratory of Landscaping Agriculture, Ministry of Agriculture and Rural Affairs, College of Horticulture, Nanjing Agricultural University, Nanjing 210095, China; wuze@njau.edu.cn

**Keywords:** strawberry, floral induction, RNA-seq, transcription factor, hormone

## Abstract

The initiation and quality of flowering directly affect the time to market and economic benefit of cultivated strawberries, but the underlying mechanisms of these processes are largely unknown. To investigate the gene activity during the key period of floral induction in strawberries, time-course transcriptome analysis was performed on the shoot apex of the strawberry cultivar ‘Benihoppe.’ A total of 7177 differentially expressed genes (DEGs) were identified through pairwise comparisons. These DEGs were grouped into four clusters with dynamic expression patterns. By analyzing the key genes in the potential flowering pathways and the development of the leaf and flower, at least 73 DEGs that may be involved in the regulatory network of floral induction in strawberries were identified, some of which belong to the NAC, MYB, MADS, and SEB families. A variety of eight hormone signaling pathway genes that might play important roles in floral induction were analyzed. In particular, the gene encoding DELLA, a key inhibitor of the gibberellin signaling pathway, was found to be significantly differentially expressed during the floral induction. Furthermore, the differential expression of some important candidate genes, such as *TFL1*, *SOC1*, and *GAI-like*, was further verified by qRT-PCR. Therefore, we used this time-course transcriptome data for a preliminary exploration of the regulatory network of floral induction and to provide potential candidate genes for future studies of flowering in strawberries.

## 1. Introduction

Strawberries (*Fragaria* sp.) belong to the large botanical family Rosaceae which contains many species with high economic value worldwide [1,2]. Most strawberry genotypes are short-day (SD) plants that are induced to flower in the autumn when exposed to decreasing photoperiod and temperature [3,4,5]. With the onset of these “autumn signals”, the shoot apical meristem (SAM) undergoes a transformation from leaf primordia to flower primordia, and the number of branch crowns (axillary leaf rosette) from the leaf axils is increased with floral induction, which may promote additional inflorescences and has a strong effect on berry yield [1,2,6,7,8,9,10]. Thus, floral induction is one of the most important stages of plant growth and development in agricultural production.

A major ecological trait of perennial plants is the timing of the transition to flowering, which determines the transition from vegetative to reproductive growth. The genetic, epigenetic, hormonal, and environmental factors for the transition from the vegetative to reproductive stage are best understood in the annual long-day (LD) plant Arabidopsis thaliana [11,12]. At least 180 genes have been shown to be involved in flowering-time control in Arabidopsis, and these genes mainly function in regulatory networks of different pathways that integrate environmental (photoperiod, vernalization, and temperature) and endogenous (autonomous, gibberellin, circadian clock, and age) signals [11,13]. *CONSTANS* (CO) is a central activator that mediates the photoperiodic pathway. *FLOWERING LOCUS C* (*FLC*) acts as a central suppressor to regulate the vernalization and autonomous pathways. The concentrations of *SQUAMOSA PROMOTER BINDING LIKE (SPL)* transcription factors increase with plant age. All of these genes regulate the downstream floral pathway integrator transcription factors *FLOWERING LOCUS T* (*FT*), *SUPPRESSOR OF OVEREXPRESSION OF CONSTANS 1* (*SOC1*), and *AGAMOUS LIKE24* (*AGL24*), which activate the expression of the meristem identity genes *LEAFY* (*LFY*), *APETALA1* (*AP1*), *SEPALLATA3* (*SEP3*), and *FRUITFULL* (*FUL*) [11,14,15]. In addition, mutations that reduce the expression of biosynthesis pathway genes or enhance the degradation of gibberellic acid (GA) showed delayed flowering [16]. The functions of these homologous genes from different species, especially model plants, are gradually becoming clear.

Flowering has always been the focus of strawberry crop research. The induction of flowers generally takes three steps: the vegetative stage, in which vegetative SAM gives rise to leaf primordia; then SAM shifts to the reproductive stage, where the initiation of flower meristem occurs from SAM and the entire shoot apex looks fat; followed by the next stage, the development of flower primordia [17,18]. Although *TERMINAL FLOWER 1* (*TFL1*) functions to maintain the vegetative meristem in both the woodland strawberry (*Fragria vesca*) and Arabidopsis, it has been shown to be the major floral suppressor in the strawberry, whereas it plays only a minor role in flowering time in Arabidopsis [19,20,21,22]. According to previous studies, *FvTFL1* integrates both photoperiodic and temperature signals to control floral induction in strawberries. Weak expression of *FvTFL1* in cool temperatures below 13 °C or SDs at temperatures of 13–20 °C allows floral induction to occur [21,23,24]. Photoperiodic flowering has been better studied than temperature-induced flowering in *F. vesca*. The activation of FvCO is affected by light, and CO plays a major role in activating *FvFT1* in leaves. FT acts as a florigen, moving to the shoot apex to form leaves, and SOC1 acts as the hub for multiple flowing times in Arabidopsis. However, the leaf-expressed *FvFT1* in strawberries represses flowering by activating *FvTFL1* at SAM through FvSOC1 in LDs [13,20,21,25]. Whereas under SD, the silenced FvFT1-FvSOC1-FvTFL1 pathway leads to the increased expression of the floral meristem identity marker genes *FvAP1* and *FvFUL1*, which were considered a floral marker gene and have been used many times in the studies of strawberry flowering [21,25,26,27,28]. Xiong, et al. [29] reported that *FvSPL10*, the ortholog of *AtSPL9* in Arabidopsis, activates *FvAP1* by directly binding to its promoter and ectopic expression of *FvSPL10* in Arabidopsis promotes early flowering and increases organ size. However, whether *FvSPL10* is involved in regulating the age pathway in strawberry flowering is unknown at present.

Axillary buds (AXBs) fate is closely related to SAM fate in strawberries. AXBs mainly differentiate into runners (a type of elongated branch) during vegetative growth. In contrast, in reproductive growth, runner induction ceases, and the number of branch crowns increases with floral induction [1,2,6,7,8,9,10]. As a result, a trade-off between flowering and runner formation has been proposed, and it is also obvious in perpetual flowering strawberry varieties, in which flowering is promoted by LD (long days), but very few runners are produced [1,7,8,9,10]. GA is one of the key factors involved in floral induction and AXB differentiation in strawberries [7]. GA3 treatment can effectively inhibit floral induction and promote runner formation in strawberries [30,31,32]. Mutations in the rate-limiting enzyme gene *GA20ox4* in the GA biosynthetic pathway result in a runner-free phenotype. Additionally, a mutation in a *DELLA* growth inhibitor gene that regulates the GA signaling pathway caused a runner-less phenotype [27,33,34]. In addition, the floral integrator SOC1 affects both floral induction and AXB fate by regulating the GA synthesis pathway, which provides evidence for GA inhibition of strawberry floral initiation [25]. However, the effect of GA on floral induction varies with species. GA can promote flower formation in Arabidopsis but inhibit flowering in apples and strawberries [7,35,36].

Although several genes that control flowering in strawberries have been identified and functionally characterized, more details of the floral induction of this important berry crop have not been explored. In this study, we generated time-course RNA-seq data of the three floral induction stages in the cultivated strawberry ‘Benihoppe.’ Many genes potentially related to the floral induction process were investigated, such as the genes for seven potential flower-time control pathways, leaf development, and flower development during floral induction. In addition, the MADS family and SEB family genes, closely related to flowering, and eight hormone signaling pathway genes were investigated. These transcription factors and a variety of hormone signals form a regulatory network to regulate floral induction. These results will provide new ideas for future functional studies and shed light on the genetic control of floral induction in strawberries.

## 2. Results and Discussion

### 2.1. Morphological Observation and FaAP1 Expression during Floral Induction in ‘Benihoppe’ Strawberry

**Figure 1:**
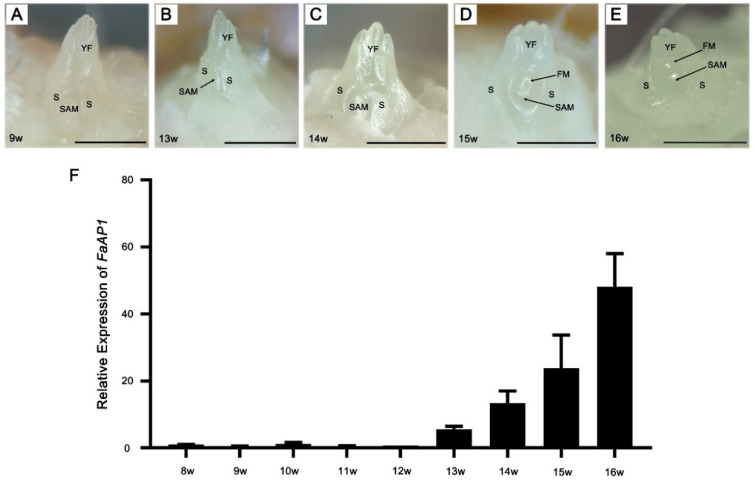
Overview of floral induction in strawberry ‘Benihoppe.’ (**A**–**E**) Morphological observation of the shoot apices at different time points during the induction of flower in strawberry. The observation results of 9 w, 13 w, 14 w, 15 w, and 16 w are shown (**A**–**E**). SAM, shoot apical meristem; S, Stipule; YF, young leaf; FM, flower meristem. Scale bar = 1 mm. (**F**) QRT-PCR analysis of the expression of the flowering marker gene *FaAP1* from 8 w to 16 w. Data are displayed as averages ± SD of three biological repeats.

The daughter plants of ‘Benihoppe’ were rooted on 6 June 2018, which was recorded as the 0 week (0 w) (Figure S1). In order to understand the morphological changes and corresponding marker genes of the strawberries during the floral induction, samples were collected weekly from 8 weeks (8 w), and the morphology of the SAMs was observed. We found that SAMs showed no significant changes from 8 w to 13 w and remained in the vegetative stage (Figure 1A,B). Then the SAM looked fatter from 14 w (Figure 1C), it was speculated that the flower meristem was starting, and the SAM shifted to the reproductive stage. Further, the flower primordia could be observed from 15 w with the changes in the environment (Figure 1D,E). By analyzing the expression of the *FaAP1* during 8 w–16 w, the results showed that expression gradually increased from 13 w, suggesting that *FaAP1* expression is up-regulated prior to the morphological change (Figure 1F).

### 2.2. Transcriptome Sequencing

Considering the results of the morphological observations and the expression profile of *FaAP1*, samples at 9 w (vegetative stage), 13 w (shifts to the reproductive stage at gene level), and 15 w (reproductive stage) were selected for transcriptome sequencing. A total of 9 cDNA libraries were prepared from the RNA extracted from the shoot apex. After Illumina sequencing, the data were filtered to remove low-quality reads and adapters. A total of 441,229,076 clean reads were obtained, of which the clean base was 66.18 G, and the average GC content was 47.61%. An average of 98.36% of the sequenced bases had quality scores (Q-scores) of Q30 or higher (Table 1). Clean reads from the nine libraries were compared with the *Fragaria* × *ananassa* ‘Camarosa’ Genome (Version 1.0).

Spearman correlation coefficient analysis showed that the correlation coefficients of the three replicates were greater than 0.925, indicating that the RNA-seq data could be used for further analysis (Figure S2).

### 2.3. GO Category Enrichment Analysis of the Differentially Expressed Genes

|Log2 fold-change| (|Log2 FC|) ≥1, and *p* value < 0.05 were used as the critical thresholds to define the differentially expressed genes (DEGs). When compared with plants at 9 w, 3719 DEGs were significantly changed at 13 w, and of these, 2368 DEGs were up-regulated, and 1351 DEGs were down-regulated. Compared with plants at 13 w, 4525 genes showed significant differential expression at 15 w; 2174 DEGs were up-regulated, and 2351 DEGs were down-regulated (Figure 2A). Based on the expression trend of the DEGs, 322 DEGs that were up-regulated at both 13 w and 15 w were classified as Cluster 1 (Figure 2B,C), and 18 DEGs that were down-regulated at both 13 w and 15 w were classified as Cluster 2 (Figure 2B,D). The 463 DEGs that were up-regulated at 13 w and down-regulated at 15 w were classified as Cluster 3 (Figure 2B,E), and 264 DEGs that were down-regulated at 13 w and down-regulated at 15 w were classified as Cluster 4 (Figure 2B,F). To explore the functions of the genes in the four clusters of DEGs, we performed GO category enrichment analysis, and the results showed that the DEGs in cluster 1 were concentrated in the ‘biological process’ GO category in terms such as ‘cell division,’ ‘nucleus,’ ‘DNA binding,’ and ‘protein binding function’ (Figure 2G). The DEGs in cluster 2 were concentrated in the terms ‘response to auxin,’ ‘regulation of transcription,’ ‘nucleus,’ and ‘DNA binding transcription factor activity functions’ (Figure 2H). The genes in cluster 3 were mainly concentrated in the ‘regulation of transcription,’ ‘nucleus,’ and ‘molecular functions’ terms (Figure 2I), and the genes in cluster 4 were mainly concentrated in the terms ‘chloroplast, ‘cytoplasm’, ‘protein binding’, and ‘oxidation-reduction process’ (Figure 2J). These results are a preliminary analysis of the clustering of differentially expressed genes. In addition, to further explore the high-expression (FPKM>2) of DEGs at the three time points during the floral induction in the strawberries, |Log2FC| ≥ 2, *p* < 0.001, and average of FPKM>2 was used as the critical thresholds to define the highly differentially expressed genes (HDEGs). The functions of unknown genes or the unknown functions of known genes can be identified by clustering genes with the same or similar expression patterns. Hierarchical clustering of the HDEGs, which were clustered by the log2 of the FPKM values, is shown in Figures S3–S5.

### 2.4. Comparison of Potential Flowering Pathway Genes in Strawberry and Arabidopsis

To fully identify candidate genes in the flowering time pathway in the strawberries, we retrieved 68 homologs of important Arabidopsis flower induction genes from the transcriptome data and analyzed their FPKM values. These genes are roughly classified as participating in the photoperiod, vernalization, autonomous, gibberellin, age, and carbohydrate pathways based on the known flowering pathways in Arabidopsis [1,11,13].

The FPKM values of some genes that are involved in the photoperiod pathway can be detected in the shoot apex. Surprisingly, some DEGs in this pathway that appear to have inconsistent functions were identified in short-day strawberries as compared with long-day Arabidopsis (Figure S6). For example, *FLAVIN BINDING KELCH REPEAT F-BOX 1* (*FKF1*) [37], *GIGANTEA* (*GI*) [38], *PSEUDO-RESPONSE REGULATOR 5* (*APRR5*), and *APRR7* [39] play positive roles in flowering in Arabidopsis, while the expression of their strawberry homologs was significantly decreased at 13 w compared with 9 w (Figure S6), even though *CO* and *FKF1* were both promoted in the leaves during the blue light-induced floral induction of strawberry [40]. However, strawberry homologs of *LUX ARRHYTHMO* (*LUX*) [41], *NUCLEAR FACTOR Y*, *SUBUNIT B2* (*NF-YB2*) [42], and *CONSTITUTIVE PHOTOMORPHOGENIC 1-like* (*COP1-like*) [43] showed similar expression trends during the floral induction compared with Arabidopsis genes. For this result, we speculated that these genes are mainly expressed and function in leaves. Thus, their transcript level in the SAM is not very meaningful for functional analysis of these genes during the floral induction.

**Figure 3:**
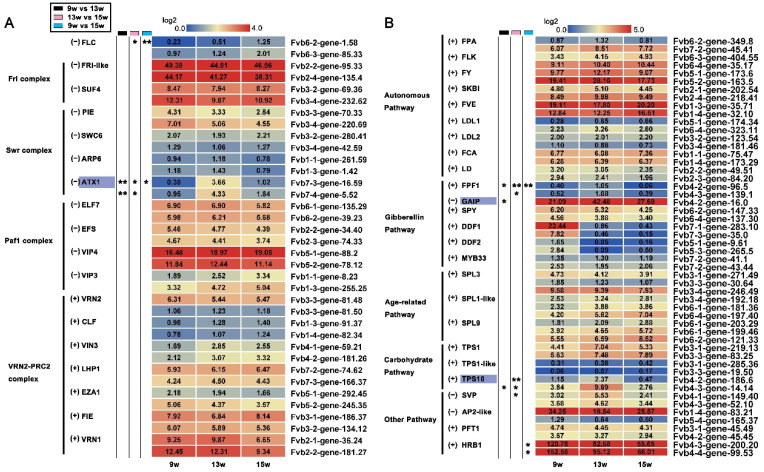
FPKM values of key genes belonging to the different flowering time pathways. (**A**) Heat map showing the expression of genes involved in the vernalization pathway. (**B**) Heat maps showing the expression of genes involved in the autonomous, GA, age-related, carbohydrate, and other pathways. (+) indicates the positive regulators of flower bud differentiation in Arabidopsis, and (−) indicates negative regulator. 9 w: vegetative stage, 13 w: shifts to reproductive stage at gene level, 15 w: reproductive stage. The column with the black boxes represents the student’s *t* test analysis of DEGs at 9 w vs. 13 w; The column with the pink boxes represents the student’s *t* test analysis of DEGs at 13 w vs. 15 w; The column with the blue boxes represents the student’s *t* test analysis of DEGs at 9 w vs. 15 w. (* |Log2FC| ≥ 1, *p* < 0.05. ** |Log2FC| ≥ 2, *p* < 0.01).

In addition to the photoperiod pathway, the vernalization pathway is also an important flowering pathway in Arabidopsis. In this transcriptome data, only *FLOWERING LOCUS C* (*FLC*) [44] and *ARABIDOPSIS TRITHORAX 1* (*ATX1*) [45] were identified as DEGs among the homologous genes related to the vernalization pathway (Figure 3A). Although both *FLC* and *ATX1* act repressors during flowering in Arabidopsis, our results show that the expression of *FLC* at 15 w was significantly increased compared to 9 w, and the expression of *ATX1* was increased at 13 w and decreased at 15 w. It is speculated that in addition to the factors of the expression locations of some genes, changes in seedling status and environment may also be significant factors.

We did not find DEGs in the autonomous and age-related pathways in the strawberries (Figure 3B). Although homologs of these genes have been reported to play important roles in flowering in Arabidopsis or other species [1,13,46], no significant changes were found during the floral induction in our RNA-seq data. As to the gibberellin pathway, *FLOWERING PROMOTING FACTOR 1* (*FPF1*) [47], *DWARF AND DELAYED FLOWERING 1* (*DDF1*) [48], and *DDF2* showed a declining trend in general, while they are promoters of flowering in Arabidopsis. In addition, *GAIP* [49], an inhibitor of the gibberellin signaling pathway, was upregulated at 13 w, although it acts as a suppressor in Arabidopsis flowering (Figure 3B). These results suggest that they might play opposite roles to Arabidopsis in the process of floral induction in strawberries. Carbohydrate is also thought to play an important role in regulating flowering. We found no significant difference in the expression of *TREHALOSE-6-PHOSPHATE SYNTHASE 1* (*TPS1*) [50], but *TPS10* was upregulated at 13 w and was down-regulated at 15 w. In other pathways, we detected continuous down-regulation of *HYPERSENSITIVE TO RED AND BLUE 1* (*HRB1*) [51] (Figure 3B). Taking all of these results into consideration, the functions of key flowering genes in Arabidopsis might not be conserved in strawberries, especially *GAIP* encoding the DELLA protein, which can be further discussed.

### 2.5. Analysis of DEGs with Roles in Leaf Development and Flowering

**Figure 4:**
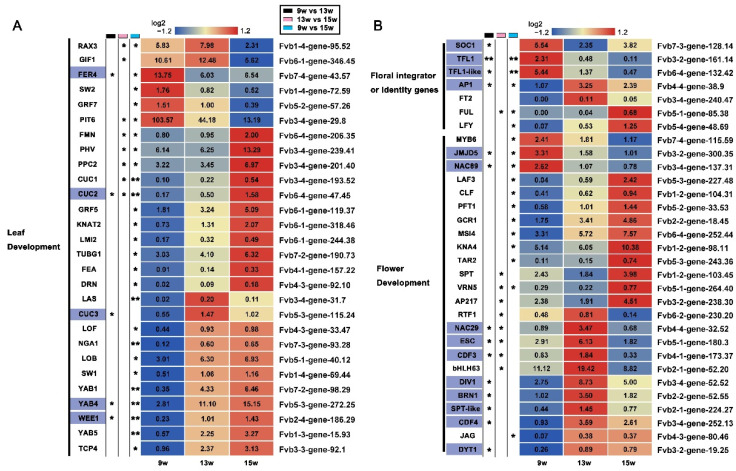
FPKM values of DEGs related to leaf and flower development. (**A**) Heat map showing the DEGs that may be involved in leaf development. (**B**) Heat map showing the DEGs that may be involved in flower development. The purple boxes indicate the genes in which expression was significantly changed at 13 w. 9 w: vegetative stage, 13 w: shifts to reproductive stage at gene level, 15 w: reproductive stage. The column with the black boxes represents the student’s *t* test analysis of DEGs at 9 w vs. 13 w; The column with the pink boxes represents the student’s *t* test analysis of DEGs at 13 w vs. 15 w; The column with the blue boxes represents the student’s *t* test analysis of DEGs at 9 w vs. 15 w. (* |Log2FC| ≥ 1, *p* < 0.05. ** |Log2FC| ≥ 2, *p* < 0.01).

Compared with the sample containing only SAM and a young leaf at 9 w, the sample at 13 w consists of SAM, a young leaf, and the flower meristem in the beginning, and the sample at 15 w consists of SAM and flower primordia (Figure 1A–E). Thus, investigating DEGs related to leaf development and flower development may help better understand the regulatory network of floral induction [18,52]. Expression patterns of 28 homologs of leaf development genes were investigated, including strawberry homologs of growth-regulating factor *GRF*, cell proliferation gene *GRF1-INTERACTING FACTOR* (*GIF1*), adaxial-adaxial gene *PHAVOLUTA* (*PHV*) and some active family members, such as *REGULATOR OF AXILLARY MERISTEMS 3* (*RAX3*), *KNOTTED-LIKE FROM ARABIDOPSIS THALIANA 2* (*KNAT2*), and *LATERAL ORGAN FUSION 1* (*LOF*) genes of MYB family, *CUC1/2/3* of NAC family, and *YAB1/5* and *YAB4-like* genes of YABBY family (Figure 4A). Compared with the DEGs of leaf development in diploid strawberries, several conserved genes were detected, such as *LATERAL SUPPRESSOR* (*LAS*), *CUC2/3*, *FASCIATED EAR* (*FEA*), etc. [18], which can serve as key genes for studying leaf development during the floral induction of strawberry.

Floral induction is a prerequisite for flower development. It has been shown that the floral integrator FvSOC1 acts as a repressor by promoting *FvTFL1* during the floral induction in short-day strawberries [25]. In this data, the significant reduction in the expression of *SOC1* and *TFL1* in our transcriptome at 13 w further verified the repressor role of *SOC1* (Figure 4B). The homologs of FT in the strawberry *FvFT1* were not detected in the RNA-seq data because it is only expressed in old leaves [18,21]. However, as another homolog of the FT, *FT2* was detected in the shoot apices, and its expression was up-regulated at 13 w, indicating that *FT2* may function differently from *FT1*. By analyzing the expression of flower identity genes, we found that the expression of *LFY*, *AP1*, and *FUL* continuously increased as expected, indicating the reliability of the samples. These genes were conserved during the floral induction of octoploid strawberry and diploid strawberry [18,21,25]. Further, several homologs of flower development genes are shown in Figure 4B. The number of the NAC family, MYB family, and bHLH family members accounted for 12.5%, 8.3%, and 16.7% of the 24 DEGs, respectively. They were *BRUNO-LIKE 1* (*BRN1*), *NAC29,* and *NAC89* of NAC family; *bHLH63*, *DYSFUNCTIONAL TAPETUM 1* (*DYT1*), *SPATULA* (*SPT*), and *SPT-like* of bHLH family; *DIVARICATA 1* (*DIV1*) and *MYB6* of MYB family. Besides, primordia-specific gene such as *JAGGED* (*JAG*) was also explored. These genes can also be considered candidate genes for studying flower development in strawberries.

### 2.6. Expression Patterns of DEGs from the MADS and SEB Families

**Figure 5:**
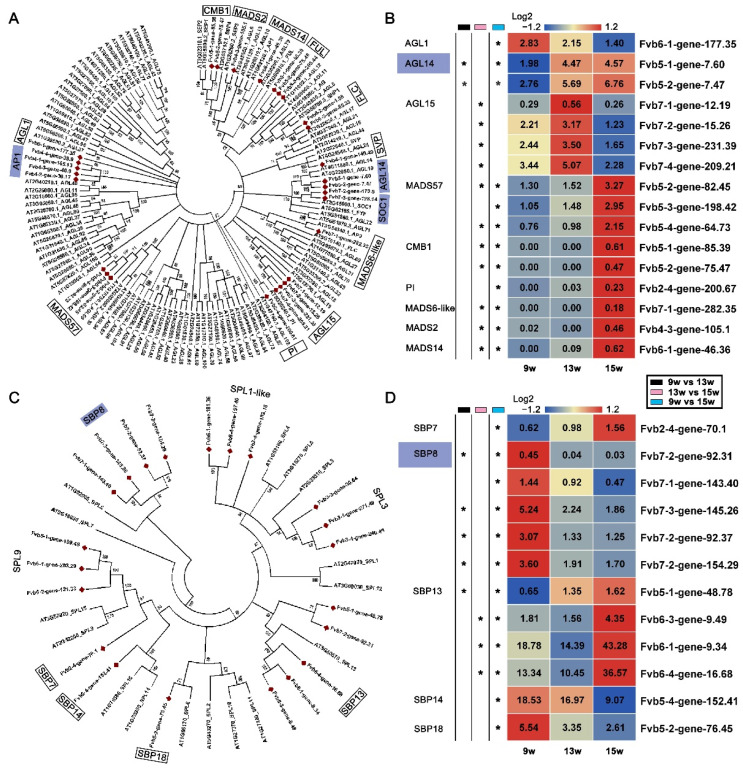
Phylogenetic analyses and relative expression of the DEGs in the MADS and SEB families. (**A**) Phylogenetic analysis of DEGs in the MADS family. (**B**) Heatmap showing the relative expression of DEGs in the MADS family. (**C**) Phylogenetic analysis of DEGs in the SEB family. (**D**) Heatmap showing the relative expression of DEGs in the SEB family. The purple boxes indicate the genes in which expression was significantly changed at 13 w. 9 w: vegetative stage; 13 w: shifts to reproductive stage at gene level; 15 w: reproductive stage. The column with the black boxes represents the student’s *t* test analysis of DEGs at 9 w vs. 13 w; The column with the pink boxes represents the student’s *t* test analysis of DEGs at 13 w vs. 15 w; The column with the blue boxes represents the student’s *t* test analysis of DEGs at 9 w vs. 15 w. (* |Log2FC| ≥ 1, *p* < 0.05).

In our analysis of the genes involved in flowering pathways, as well as floral integrators and identity genes, we found at least four MADS family members—*AP1*, *FLC*, *SOC1,* and *SVP* (Figure 4B). This indicated that MADS family genes might play an important role in the process of flowering in strawberries. MADS family members in cotton, wintersweet (*Chimonanthus praecox*), peach, pear, and Arabidopsis were also found to be involved in several plant growth and development processes, such as flower morphogenesis, floral induction, gibberellin synthesis, and delay of senescence [53,54,55,56,57]. To investigate the expression of other MADS family members in flowering in strawberries, the nine different genes and their copies were identified by comparing the MADS family in strawberry with the MADS family in Arabidopsis. They are *AGAMOUS-LIKE 1* (*AGL1*), *AGL14*, *MADS57*, *AGL15*, *CMB1*, *PISTILLATA* (*PI*), *MADS6-like*, *MADS2*, and *MADS14*. Except for *AGL14* and *AGL15*, which were significantly up-regulated at 13 w, the other genes, such as *MADS57* and *CMB1*, mainly showed significant changes in expression at 15 w (Figure 5A,B), indicating that most MADS family members could have roles in the reproductive stage.

The SBP family has been reported to be a plant-specific family whose members play important roles in early flowering [58], nutritional change and reproductive stages [59], and the gibberellic acid response [60]. We found that the three SBP family members, *SPL3*, *SPL1-like*, and *SPL9*, showed no significant expression changes during floral induction based on our data, although these homologs were previously reported to be involved in the age pathway of flowering in Arabidopsis [61]. To understand the expression patterns of other members of the SBP family in strawberries, we performed phylogenetically and expression analyses (Figure 5C,D). The results showed that SBP8 and SBP18 were significantly down-regulated at 13 w. In comparison, SBP13 and SBP7 were significantly up-regulated at 15 w, and SBP14 was significantly down-regulated at 15 w compared with expression at 9 w (Figure 5C,D). These results suggest that these SBP family genes might also respond to the process of floral induction in strawberries.

### 2.7. Expression Analysis of DEGs in Different Hormone Signaling Pathways

**Figure 6:**
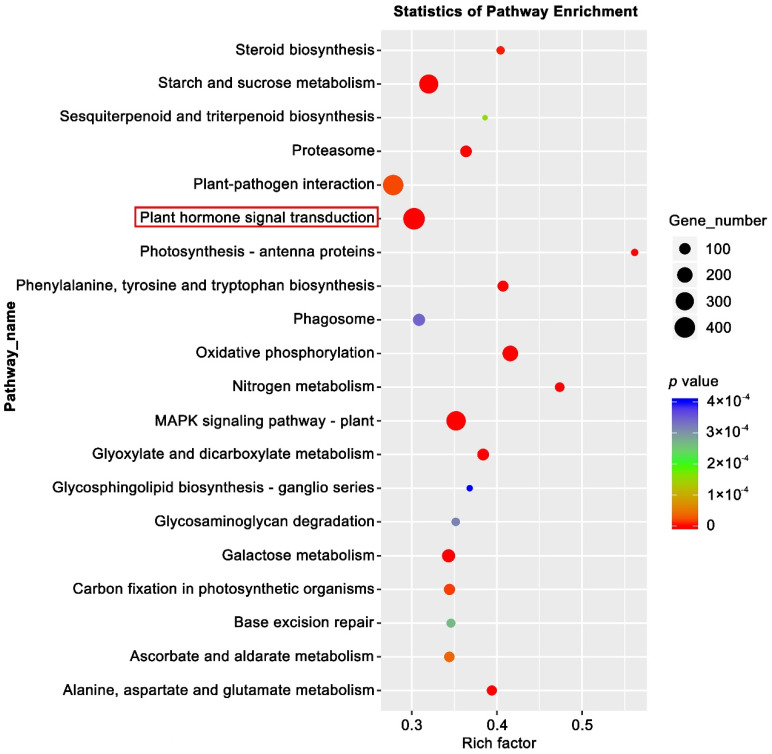
Kyoto Encyclopedia of Genes analysis of DEGs. KEGG pathway analysis of the DEGs showing the top 20 enriched pathways. The most enriched pathway—Plant hormone signal transduction is indicated by the red box.

**Figure 7:**
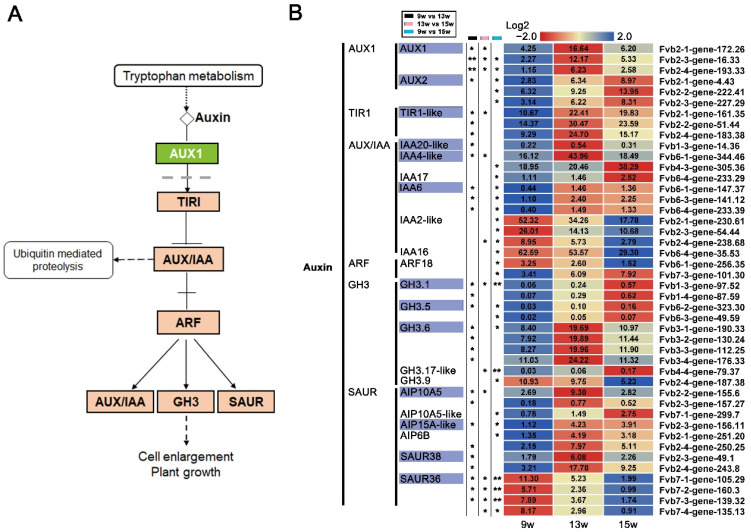
DEGs involved in auxin signaling pathways. (**A**) Auxin signal transduction pathways by referring to the KO04075 pathway in KEGG enrichment analysis. (**B**) Heatmaps showing the relative expression of DEGs involved in the auxin. The purple boxes indicate the genes in which expression was significantly changed by 9 w vs. 13 w. 9 w: vegetative stage, 13 w: shifts to reproductive stage at gene level, 15 w: reproductive stage. The column with the black boxes represents the student’s *t* test analysis of DEGs by 9 w vs. 13 w. The column with the pink boxes represents the student’s *t* test analysis of DEGs by 13 w vs. 15 w; The column with the blue boxes represents the student’s *t* test analysis of DEGs by 9 w vs. 15 w. (* |Log2FC| ≥ 1, *p* < 0.05. ** |Log2FC| ≥ 2, *p* < 0.01).

Plant hormones, including auxin, cytokinin (CK), abscisic acid (ABA), GA, ethylene, brassinosteroids, jasmonic acid (JA), and salicylic acid (SA), play a synergistic role in floral induction in response to external stimulation and the local environment [62]. KEGG enrichment analysis of the DEGs showed that the most were enriched in the plant signal transduction pathways (Figure 6). Therefore, by referring to the KO04075 pathway (https://www.kegg.jp/kegg-bin/show_pathway?ath04075 accessed on 2 March 2022), the changes in the expression of key genes in various hormone signal transduction pathways during strawberry floral induction were explored. In the RNA-seq data, most of the key genes in the auxin signal transduction pathway were up-regulated (Figure 7A,B). *AUX1*, *TIR1-like*, *IAA20-like*, *IAA6*, *GH3.6*, *AIP10A5*, *AIP15A-like*, *AIP6B*, and *SAUR38* were significantly up-regulated at 13 w, while *AUX2*, *IAA4-like*, *GH3.1*, *GH3.5*, and *GH3.17-like* were up-regulated at 15 w, suggesting that auxin signaling plays a role in promoting floral induction in general. These genes can be used as candidate genes for further study of how flowering is related to auxin. In addition, *IAA2-like*, *GH3.9*, and *SAUR36* were significantly down-regulated at 13 w, indicating that these genes might act as suppressors in the auxin mediated-flowering in strawberries.

**Figure 8:**
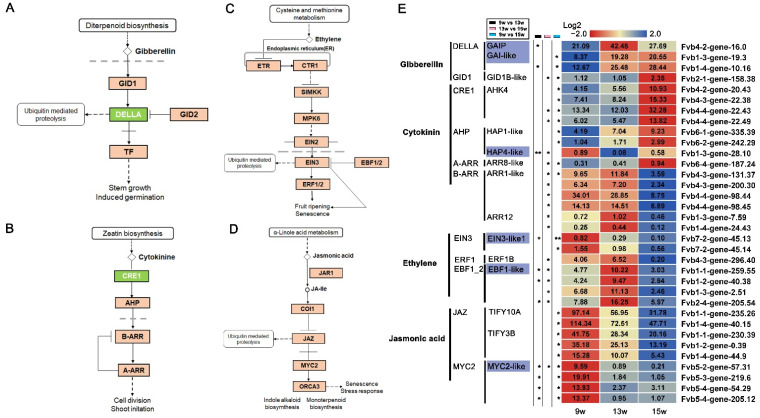
DEGs involved in gibberellin, cytokinin, ethylene, and jasmonic acid signaling pathways. (**A**–**D**) Gibberellin, cytokinin, ethylene, and jasmonic acid signal transduction pathways by referring to the KO04075 pathway in KEGG enrichment analysis. (**E**) Heatmaps showing the relative expression of DEGs involved in the above-mentioned hormone signaling pathways. The purple boxes indicate the genes in which expression was significantly changed by 9 w vs. 13 w. 9 w: vegetative stage, 13 w: shifts to reproductive stage at gene level, 15 w: reproductive stage. The column with the black boxes represents the student’s *t* test analysis of DEGs by 9 w vs. 13 w; The column with the pink boxes represents the student’s *t* test analysis of DEGs by 13 w vs. 15 w; The column with the blue boxes represents the student’s *t* test analysis of DEGs by 9 w vs. 15 w. (* |Log2FC| ≥ 1, *p* < 0.05. ** |Log2FC| ≥ 2, *p* < 0.01).

Based on the importance of GA in floral induction, the DELLA protein in the GA signaling pathway has become a popular focus of research. It is the intersection of various hormonal pathways [7,27,33,34,62]. In our data, the repressor of the GA signaling pathway, *DELLA*, was significantly up-regulated at 13 w. At the same time, GID1B-like, the gibberellin receptor and the inhibitor of *DELLA*, was decreased at 13 w (Figure 8A,E), indicating that GA signaling might inhibit floral induction in strawberries, contrary to its role in floral induction in Arabidopsis.

*CRE1* and *AHP* are both promoters in the cytokinin signaling pathway (Figure 8B). Our transcriptome data showed that the expression of both genes did not change significantly at 13 w but began to be significantly up-regulated until 15 w (Figure 8E). In the downstream part of the cytokinin pathway, *A-ARR* expression was increased at 15 w, which was consistent with that of the upstream part. Additionally, expression of *B-ARR*, which is negatively regulated by *A-ARR*, was opposite to that of *A-ARR*. These results suggest that the cytokinin signal transduction pathway might mainly play a role in the reproductive stage. In cytokinin-mediated floral induction in Arabidopsis, DELLA was reported to bind B-ARR, which induces DELLA to re-target the promoter regulated by cytokinin for activation [63,64]. Combined with the analysis of *DELLA* expression, our results showed that GA and cytokinin might have antagonistic effects on strawberry floral induction.

In the ethylene signaling pathway, the repressor EBF1/2 further inhibits *ERF1* by directly inhibiting *EIN3*, ultimately affecting ethylene signal transduction (Figure 8C). In Arabidopsis, EIN3 delays flowering by activating *ERF1* in the APETALA2 (AP2)/ERF1 protein family and its evolutionary relatives, and there is a link between ethylene signaling and DELLA, that the inhibition of EIN3-ERF1 is mainly attributed to the decreased GA level and the accumulation of DELLA protein [65,66,67]. In our transcriptome data, we found that *EBF1-like* was up-regulated at the early stage of floral induction. Correspondingly, *EIN3-like1* was down-regulated at 13 w, and the expression of *ERF1B* was significantly decreased at 15 w (Figure 8C). This suggests that ethylene signals might also negatively regulate floral induction in strawberries. However, *DELLA* expression was up-regulated during this period, which appears to be inconsistent with the regulatory relationship between DELLA and EIN3-ERF1 during Arabidopsis floral initiation. Interestingly, it is also reported that DELLA inhibits ethylene signaling by binding EIN3 and ERFs to form a feedback regulatory network in response to ethylene signals [14,68]. We speculate that the crosstalk between DELLA and the ethylene signal may be more complicated due to the particularity of DELLA in the floral initiation process of strawberries, which should be further explored.

Jasmonic acid (JA) plays an important role in a variety of plant diseases and is involved in a variety of developmental processes, including flowering time in Arabidopsis [62]. DELLA protein can interact with JASMONATE-ZIM-DOMAIN PROTEINs (JAZs) to reduce the inhibition of its key target *MYC2* [69]. Here, significant down-regulation of *MYC2* expression suggested that the JA signal might play a negative role in floral induction. The genetic link between DELLA and JA signaling could be a point for future studies on strawberries (Figure 8D,E).

**Figure 9:**
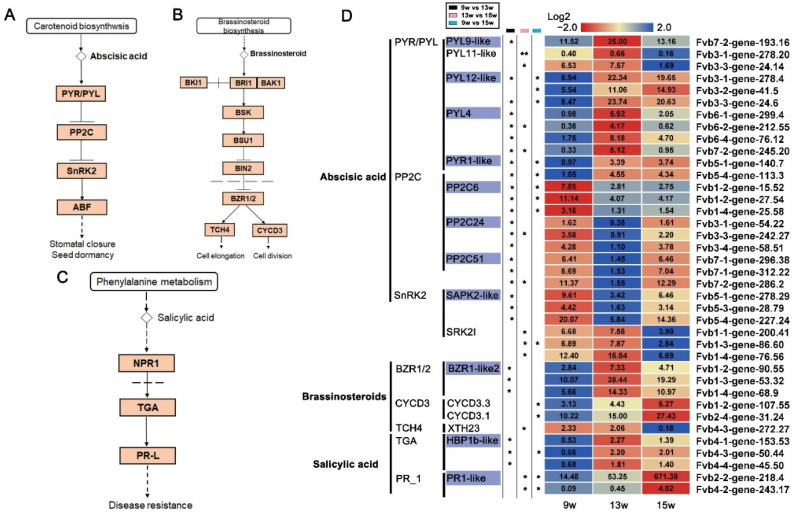
DEGs involved in abscisic acid, brassinosteroid, and salicylic acid signaling pathways. (**A**–**C**) Abscisic acid, brassinosteroid, and salicylic acid signal transduction pathways by referring to the KO04075 pathway in KEGG enrichment analysis. (**D**) Heatmaps showing the relative expression of DEGs involved in the above-mentioned hormone signaling pathways. The purple boxes indicate the genes in which expression was significantly changed by 9 w vs. 13 w. 9 w: vegetative stage, 13 w: shifts to the reproductive stage at the gene level, 15 w: reproductive stage. The column with the black boxes represents the student’s *t* test analysis of DEGs by 9 w vs. 13 w; The column with the pink boxes represents the student’s *t* test analysis of DEGs by 13 w vs. 15 w; The column with the blue boxes represents the student’s *t* test analysis of DEGs by 9 w vs. 15 w. (* |Log2FC| ≥ 1, *p* < 0.05. ** |Log2FC| ≥ 2, *p* < 0.01).

In the ABA signal transduction pathway, the ABA receptor gene *PYRABACTIN RESISTANCE 1-LIKE*/*PYRABACTIN RESISTANCE* (*PYL*/*PYR*) and other key genes were significantly up-regulated at 13 w. In contrast, the expression of the negative regulator of the ABA pathway, *PROTEIN PHOSPHATASE 2C* (*PP2C*), was decreased at 13 w. Although PP2C indirectly inhibits the expression of ABA-responsive genes by inhibiting *SNF1-RELATED PROTEIN KINASE 2* (*SnRK2*), the expression of *SAPK2-like*, belonging to SnRK2 proteins, was consistent with that of *PP2C*. However, the expression level of *SRK2I* was increased at the early stage of floral induction and decreased at 15 w (Figure 9A,D). These results indicate that the relationship between floral induction and the ABA signaling pathway is complex in strawberries.

Brassinosteroids (BRs) are thought to promote flowering in Arabidopsis [70]. We found that *BRASSINAZOLE-RESISTANT 1* (*BZR1*)-*like2* was significantly up-regulated at 13 w, and the expression of *CYCLIN-D3-1* (*CYCD3.1*) and CYCD3.3 was increased at 15 w. The surprise is that although *XYLOGLUCAN ENDOTRANSGLUCOSYLASE*/*HYDROLASE* (*XTH23*) is positively regulated by BZR1 (Figure 9B,D), it was down-regulated at 15 w. These results at least suggested that BRs signals play positive roles in the initiation of flower meristem. Although there are few reports describing the role of SA in plant floral induction, we also found two DEGs in the SA signaling pathway. The upstream promoter of the SA signaling pathway, *HBP1B-like*, was significantly up-regulated at 13 w. At the same time, the downstream promoter, *PR1-like*, was significantly up-regulated at 15 w (Figure 9C,D), suggesting that the SA signals might also promote floral induction in strawberries by participating in the complex plant hormone signaling pathways.

### 2.8. QRT-PCR Identification of DEGs

**Figure 10:**
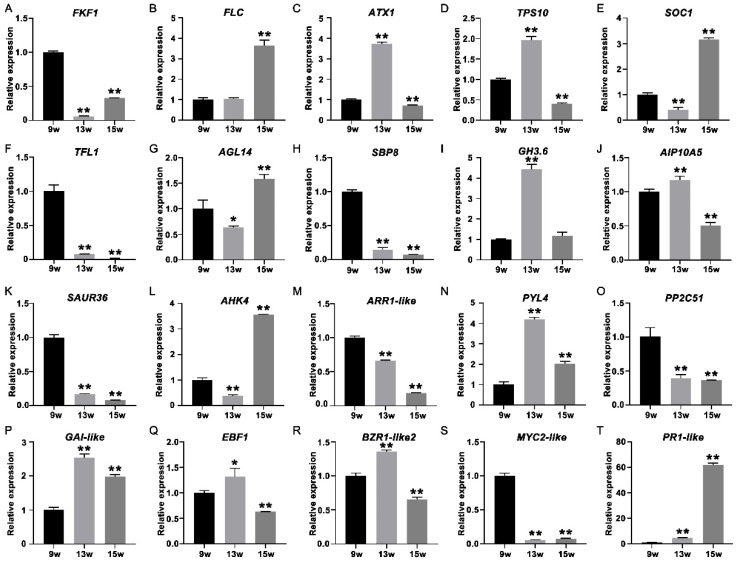
QRT-PCR analysis of 24 strawberry genes that showed differential expression at 9 w, 13 w, and 15 w to verify the RNA-seq data. (**A**–**T**) QRT-PCR identification of DEGs mentioned in the above text. Error bars indicate the standard deviation (SD) for three biological replicates. (* *p* < 0.05, ** *p* < 0.01).

To further verify the expression profile of genes determined from the RNA-seq data, 24 transcripts were selected for qRT-PCR analysis (Figure 10A–T), such as *FKF1* in the photoperiodic pathway (Figure 10A), *FLC* and *ATX1* in the vernalization pathway (Figure 10B,C), *TPS10* in the carbohydrate pathway (Figure 10D), the floral integrator *SOC1* (Figure 10E), the key repressor *TFL1* in flowering in strawberry (Figure 10F), *AGL14* in the MADS family (Figure 10G), *SBP8* in the SEB family (Figure 10H), key genes (*GH3.6*, *AIP10A5* and *SAUR36*) in the auxin signaling pathway (Figure 10I–K), two key genes (*AHK4* the *ARR1-like*) in the cytokinin signaling pathway (Figure 10L,M), *PYL4* and *PP2C51* in the ABA signaling pathway (Figure 10N,O), *GAI-like* in the GA signaling pathway (Figure 10P), *EBF1* in the ethylene signaling pathway (Figure 10Q), *BZR1-Like2* in the brassinosteroid signaling pathway (Figure 10R), *MYC2-like* in the JA signaling pathway (Figure 10S), and *PR1-like* in the salicylic acid signaling pathways (Figure 10T). These qRT-PCR results were consistent with the expression profiles obtained from the RNA-seq data, indicating the reliability of RNA-seq.

## 3. Conclusions

In the present study, 7177 DEGs that respond to floral induction signals in the shoot apex were identified in the strawberry cultivar ‘Benihoppe.’ Some key genes with conserved functions in floral initiation, leaf development, and flower development, such as *SOC1*, *TFL1*, *AP1,* and *CUC2* were detected. Two important gene families involved in floral initiation, the MADS family and SEB family, were also analyzed. We preliminarily explored the expression of key genes in eight hormone signaling pathways and believed that the *DELLA* gene could be an important candidate gene to explore the floral induction of strawberries in the future.

Currently, CRISPR/Cas9-mediated gene editing technology has been applied to strawberries [30,71], and the full use of genomics and biotechnology will help reveal genetic and epigenetic changes that can be used to improve strawberry varieties in the future.

## 4. Materials and Methods

### 4.1. Plant Material and Sample Collection

Strawberry plants (*Fragaria* × *ananassa* ‘Benihoppe’) were grown in the greenhouse in Shangzhuang Experimental Station (China Agricultural University), Beijing (S 116°23’, W 39°56’), China. The daughter plants of ‘Benihoppe’ were rooted on 6 June 2018, which we marked as the 0 week (0 w) (Figure S1). The shoot apices were sampled once weekly from 1 August 2018 (8 w) to 26 September 2018 (16 w), with three biological replicates each time, and each biological replicate contained the shoot apices of 5 plants. All collected samples were immediately frozen in liquid nitrogen and then stored at −80 °C. Sampling was performed at approximately 10 a.m. to reduce the possibility of differences in gene expression due to circadian oscillation. The temperatures and day lengths in Beijing in August and September are shown in Figure S1.

### 4.2. RNA Isolation and Qualitative and Quantitative Analysis of Total RNA

Total RNA samples were isolated from strawberry shoot apices using the E.Z.N.A. Total RNA Kit (Omega, R6834-01, Norcross, GA, USA) according to the manufacturer’s protocol. First-strand cDNAs were synthesized from total RNA using HIScript II Reverse Transcriptase (Vazyme, R233-01, Nanjing, China). The qRT-PCR reactions were performed in 10 μL volumes containing 1 μL cDNA as a template using ChamQ Universal SYBR qPCR Master Mix (Vazyme, Q711-02, Nanjing China) on an ABI Q6 Real-Time PCR System (Applied Biosystems, Foster City, CA, USA). The 2^−ΔΔCT^ method was used to analyze the qRT-PCR expression data [72]. *FaACTIN* was used as the internal control for the normalization of gene expression in strawberries. The name and sequences of gene-specific primers are given in Table S1.

### 4.3. RNA-seq

All RNA samples were delivered to Hangzhou Lianchuan Biotechnology Co. Ltd. (Hangzhou, China) to prepare cDNA libraries and high-throughput RNA sequencing on the Illumina 4000 sequencing instrument. HISAT2 software (https://ccb.jhu.edu/software/hisat/index.shtml accessed on 2 March 2022) was used for alignment, and valid reads were aligned to the reference octoploid strawberry genome (https://datadryad.org/stash/dataset/doi:10.5061/dryad.b2c58pc accessed on 2 March 2022) [73]. Splicing and merging of transcripts were performed with Stringtie software (https://ccb.jhu.edu/software/stringtie/ accessed on 2 March 2022); FPKM was used to estimate the quantification of gene expression levels. A corrected *p*-value of <0.05 and |log2foldchange| ≥1 were set as the thresholds for significant differential expression. The raw sequence data have been submitted to the NCBI Sequence Read Archive under accession number PRJNA746082.

### 4.4. Gene Expression Pattern Analysis and Functional Annotation

EdgeR software (https://bioconductor.org/packages/release/bioc/html/edgeR.html accessed on 2 March 2022), was used for transcript quantification, difference comparison, and visualization, and the differential expression results were graphically displayed using R (https://www.r-project.org/ accessed on 2 March 2022) and Tbtools (https://github.com/CJ-Chen/TBtools accessed on 2 March 2022). The GOseq R package (http://www.bioconductor.org/packages/release/bioc/html/goseq.html accessed on 2 March 2022) was used for Gene Ontology (GO) enrichment analysis, and KOBAS software was used for the Kyoto Encyclopedia of Genes and Genomes (KEGG) analysis (http://kobas.cbi.pku.edu.cn/kobas3/ accessed on 2 March 2022).

### 4.5. Shoot Apical Meristem Microscopy

Morphological observation of SAMs was carried out once a week from 8w, and five plants were sampled each time. After sampling, leaves were peeled off to expose the apical meristem step by step, and meristem morphologies were photographed using a microscope (Sz61, Olympus, Tokyo, Japan) equipped with a RisingCam industrial digital camera (E3ISPM20000KPA, Hangzhou, China).

### 4.6. Sequence Alignments and Phylogenetic Analyses

Alignments were performed using BioEdit 7.0 (https://bioedit.software.informer.com/7.0/) and ClustalW (http://www.ch.embnet.org/software/ClustalW.html accessed on 2 March 2022). Phylogenetic analyses were performed using MEGA 7.0 (https://www.megasoftware.net/ accessed on 2 March 2022). The CDS sequences of MADS family and SEB family members from Arabidopsis were downloaded from TAIR (https://www.arabidopsis.org/ accessed on 2 March 2022). The primers are listed in Table S1.

## Figures and Tables

**Figure 2:**
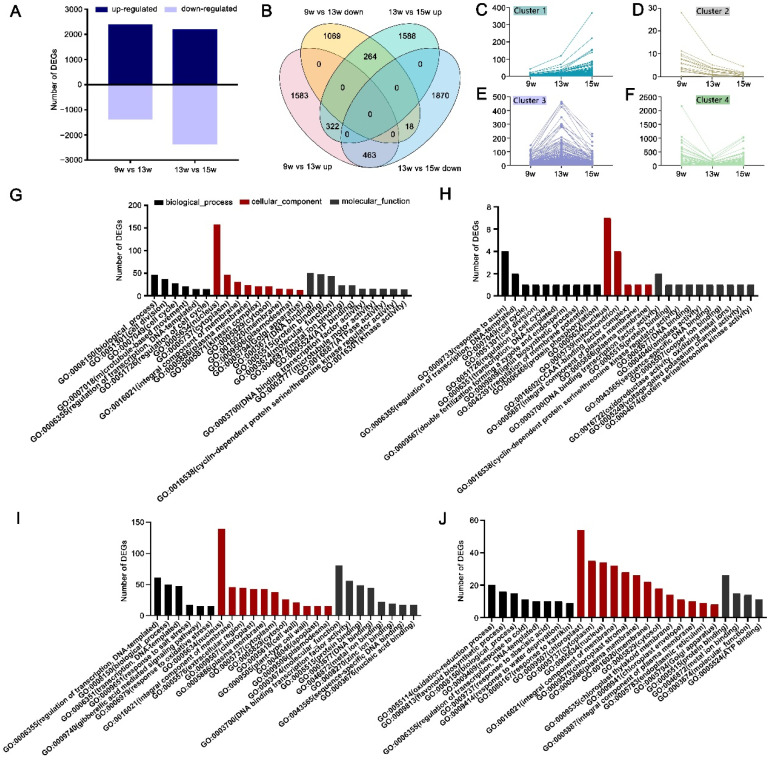
Differential gene expression and GO enrichment analysis during flowering in strawberry. (**A**) Number of up-regulated and down-regulated DEGs of 13 w and 15 w. (**B**) Venn diagram of DEGs in the 13 w and 15 w RNA-seq libraries. (**C**–**F**) Expression of the DEGs at 9 w, 13 w, and 15 w in clusters 1−4, based on the overlaps in the Venn diagram in panel B. (**G**–**J**) GO enrichment analysis of DEGs in cluster 1 (**G**), cluster 2 (**H**), cluster 3 (**I**), and cluster 4 (**J**).

**Table 1:** Overview of the RNA-seq read data from the nine strawberry flower bud libraries.

Sample	Raw Read Number ^a^	Valid Read Number	Total Mapped Reads (%)	GC Content (%) ^b^	Q30 (%) ^c^
9 w_1	54,341,278	52,680,790	89.03%	47.50	98.21
9 w_2	44,889,462	42,256,470	88.58%	47	98.18
9 w_3	51,051,002	45,602,432	90.21%	47.50	98.51
13 w_1	52,877,744	49,127,442	89.97%	48	98.46
13 w_2	54,852,944	51,190,822	90.21%	47	98.38
13 w_3	55,022,942	51,511,104	90.72%	48	98.37
15 w_1	53,068,162	49,831,302	90.95%	48	98.42
15 w_2	53,338,002	49,269,292	90.37%	47.50	98.38
15 w_3	53,002,122	49,759,422	90.52%	48	98.31

^a^: The number of reads in the original offline data; ^b^: The proportion of G and C content in four bases in valid reads; ^c^: Proportion of bases with mass value ≥ 30 (sequencing error rate less than 0.001).

## Data Availability

Not applicable.

## Supplementary Materials

The following supporting information can be downloaded at: https://www.mdpi.com/article/10.3390/ijms23116126/s1.
