# Supplementary material for: Time-Course Transcriptomic Profiling of Floral Induction in Cultivated Strawberry
Source: Int J Mol Sci. 2022 May 30;23(11):6126. doi: 10.3390/ijms23116126 (PMC9181015; doi:10.3390/ijms23116126)
Supplement: Supplementary file 1 [file ijms-23-06126-s001.zip › ijms-1705023-supplementary.pdf]

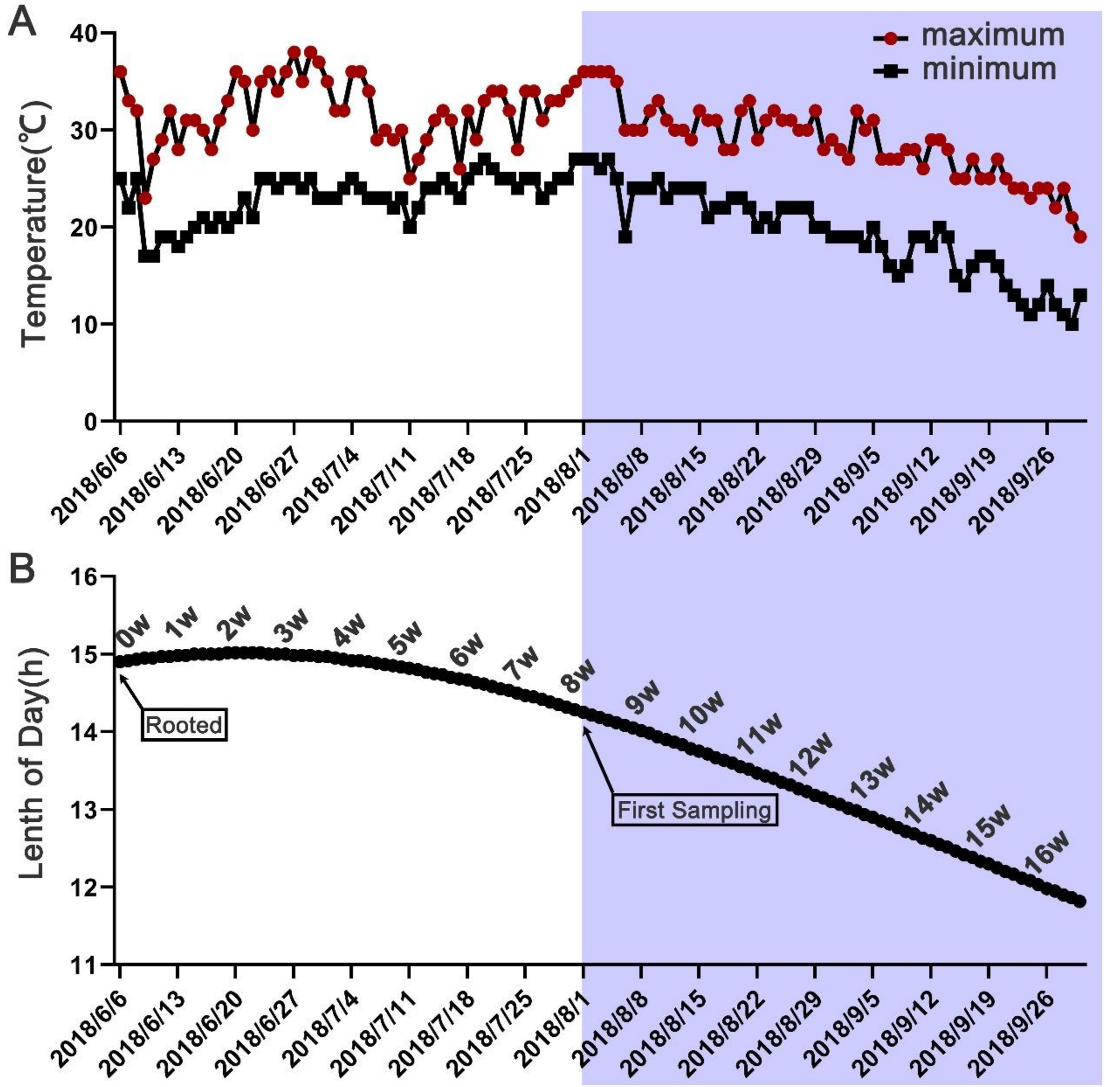

Figure S1. Temperature and daylight length in Beijing from June to September 2018. The daughter plants were rooted at 2018/6/6, which is recorded as 0w. The purple square indicates the sampling time interval, and the first time of sampling was at 8w.

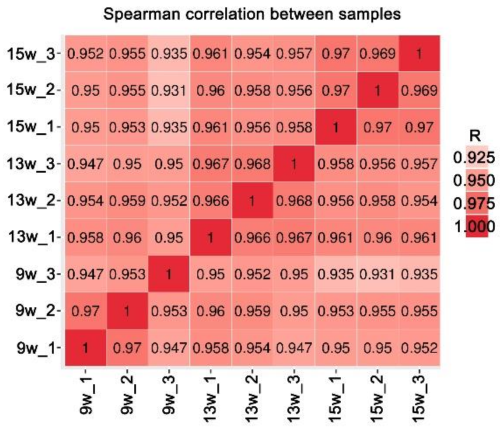

Figure S2. Hierarchical cluster diagram of gene expression levels in the nine samples.

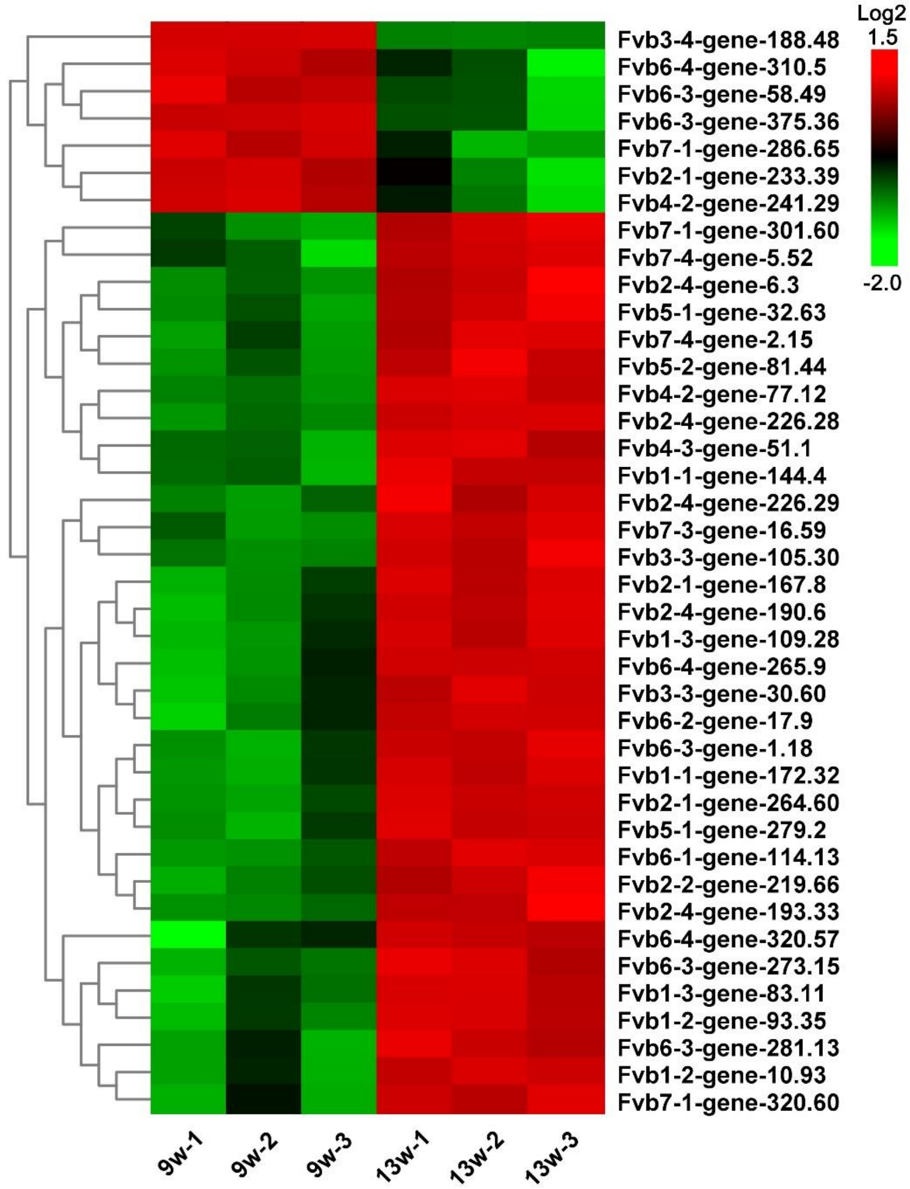

Figure S3. Heat maps of highly differentially expressed genes (|Log2FC|≥2, p <0.001) between 13w and 9w. The genes are clustered by FPKM value, with red indicating high expression genes and green indicating low expression genes.

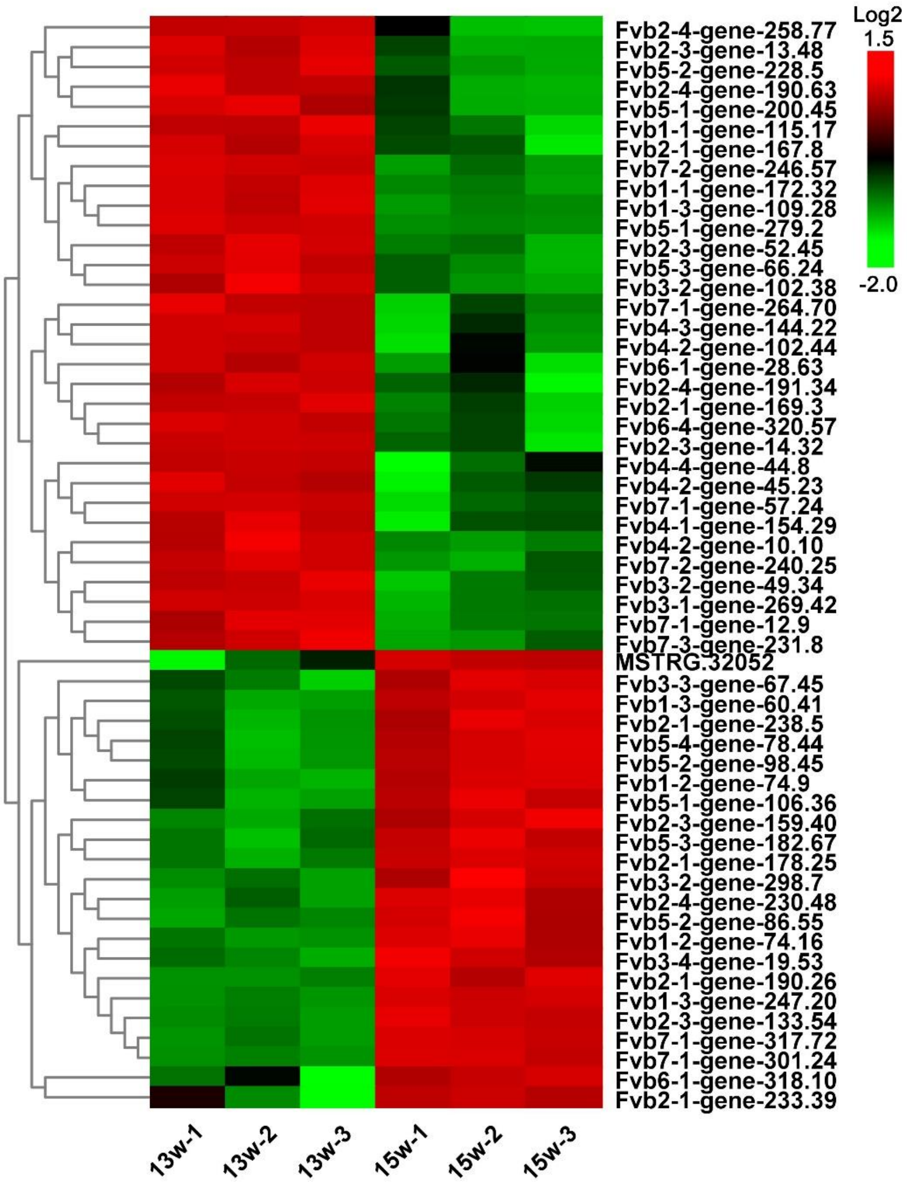

Figure S4. Heat maps of highly differentially expressed genes (|Log2FC|≥2, p <0.001) between 15 w and 13 w. The genes are clustered by FPKM value, with red indicating high expression genes and green indicating low expression genes.

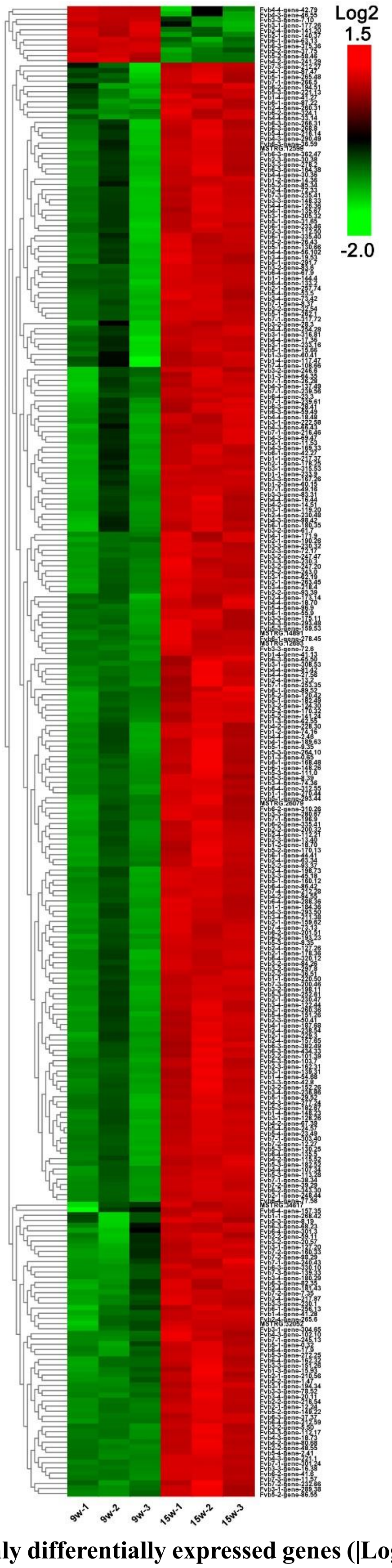

Figure S5. Heat maps of highly differentially expressed genes (|Log2FC|≥2, p <0.001) between 15 w and 9 w. The genes are clustered by FPKM value, with red indicating high expression genes and green indicating low expression genes.

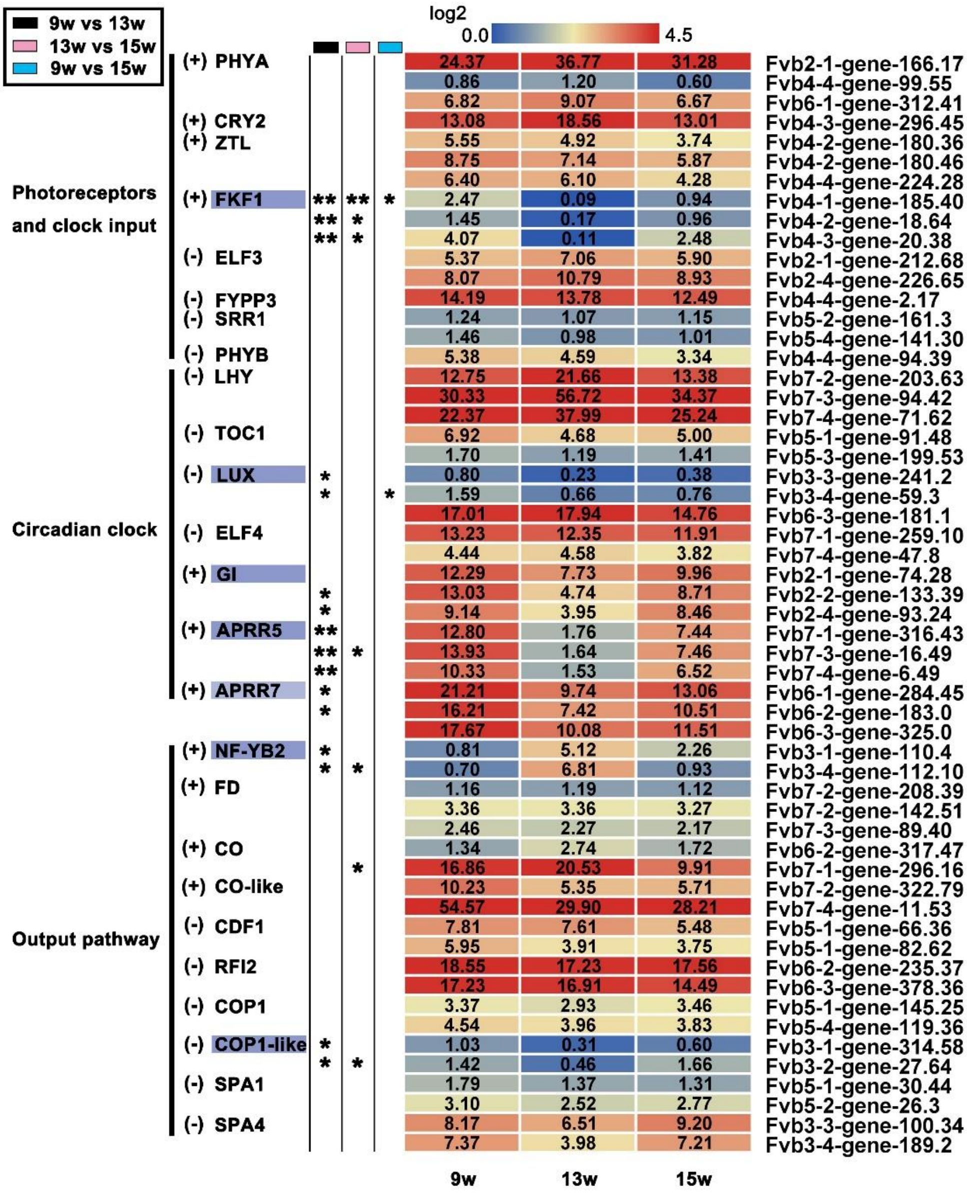

Figure S6. Heat maps showing the expression of genes involved in the photoperiod flowering pathway. (+) indicates the positive regulators of flower bud differentiation in Arabidopsis, and (-) indicates negative regulator. The purple boxes indicate the genes in which expression was significantly changed by 9w VS 13w. 9w: vegetative stage, 13w: shifts to reproductive stage at gene level, 15w: reproductive stage. The column with the black boxes represents the student's *t* test analysis of DEGs by 9w VS 13w; The column with the pink boxes represents the student's *t* test analysis of DEGs by 13w VS 15w; The column with the blue boxes represents the student's *t* test analysis of DEGs by 9w VS 15w. (* |Log2FC| ≥1, p <0.05. ** |Log2FC| ≥2, p <0.01).

Table S1. Oligonucleotide primers used in this study.

Gene ID	Gene name	Primers
Fvb7-1-gene-250.12	Actin	TGGGTTTGCTGGAGATGAT
		CAGTTAGGAGAACTGGGTGC
Fvb4-3-gene-20.38	FKF1	GCTCTGGAGCCCGATTGTCC
		GAACCGACAGTTGCGACCGA
Fvb6-2-gene-1.58	FLC	TCGTCCGCGTATGAGCAACC
		CTGGTTCTGCCCTTGGCGAT
Fvb7-3-gene-16.59	ATX1	TGCTAGCCACCGTAAGGACCA
		AGCGAACCCTAGTGAGAGCCT
Fvb4-3-gene-14.14	TSP10	GCAGCCGAGCATGGCTACTT
		AGCCATCTGTCGCCTCGGTA
Fvb7-3-gene-128.14	SOC1	CCACGAGCCGTCAAGTCACC
		ATGAGCGCAACCTCGGCATC
Fvb3-2-gene-161.14	TFL1	TGCCGAGGCCAAACATAGGG
		AAGACAGCAGCCACCGGAAG
Fvb5-1-gene-7.60	AGL14	TGCGATGCTGAGGTTGCACT
		ACCATCACCAAGGAGCTTCCG
Fvb7-3-gene-145.26	SBP8	TCTCGACCACTACGCCACCA
		CGAGGCTGAGCATGGAGTGG
Fvb3-2-gene-130.24	GH3.6	CCTTGTGGCTCGTCCAGTCC
		GCGCCAACTCGGAGGACTAC
Fvb2-2-gene-155.6	AIP10A5	ACGGCAGACGGTTCATCGTG
		GGCTGGTTGGCGAAGCCATA
Fvb7-1-gene-105.29	SAUR36	ACGGCGACTTTCACCGTGTT
		CGGGCACGGTATGGTGATCC
Fvb4-4-gene-22.43	AHK4	GAGAGGGAGCCCTCACCCAT
		GCTCCTCCACGGTTGGGTTT
Fvb4-3-gene-131.37	ARR1-like	ATGGTGGCAGAGGGACAGGA
		AGGCTGGACATCCCTTGGGT
Fvb6-4-gene-76.12	PYL4	GTGGTCCGCCGCTTCGATAA
		CAGCACGTCCAGCCTCTCAG
Fvb7-2-gene-286.2	PP2C51	TGCCGGGAGAGGTTACACGA
		CAACCAGCTCCTCCGTCACC
Fvb1-4-gene-10.16	GAI-like	CGCTTCCGACGCCGTTATCT
		ATGGCGTCAGCGAAGTCGAG
Fvb2-4-gene-205.54	EBF1	TGTCTCTTGGTCGGCGTGTG
		TGCACAGGCACTCCTCTCCT

Fvb1-3-gene-53.32	BZR1-like2	GCTGAAGCTGGTTGGGTCGT
		AGGACTGTGGGCTTGGTTGC
Fvb5-2-gene-57.31	MYC2-like	GGCCATTCCAGAGGCACCAG
		TTCCCATGGCCTGCAGCAAA
Fvb2-2-gene-218.4	PR1-like	GGGTGACGGAGAAGCCCAAC
		GCGCCAAACCACCTGGGTAT
